# Supplementary material for: A Few-Shot Learning Approach Assists in the Prognosis Prediction of Magnetic Resonance-Guided Focused Ultrasound for the Local Control of Bone Metastatic Lesions
Source: Cancers (Basel). 2022 Jan 17;14(2):445. doi: 10.3390/cancers14020445 (PMC8773927; doi:10.3390/cancers14020445)
Supplement: Supplementary file 1 [file cancers-14-00445-s001.zip › cancers-1436214.supplementary.pdf]

cancers

MDPI

Article

A Few-Shot Learning Approach Assists in the Prognosis Prediction of Magnetic Resonance-Guided Focused Ultrasound for the Local Control of Bone Metastatic Lesions

Fang-Chi Hsu, Yin-Ju Chen, Yao-An Shen, Yi-Chieh Tsai, Meng-Huang Wu, Chia-Chun Kuo, Long-Sheng Lu, Shauh-Der Yeh, Wen-Sheng Huang, Hsin-Lun Lee, Chia-Ning Shen and Jong-Fong Chiou

Supplementary Data

Sensitivity Analysis

The sensitive analysis, K-fold cross validation, was conducted to compare each splatted sub-dataset into k-subsets. Here, k-fold means the number of subsets. Our final model was used for model testing and called as the first fold. The parameters which selected by final model has already trained in our training dataset. Then, each fold of subsets was applied for model testing. The results of k-fold cross validation with k = 1, 3, and 10 is shown below. Our final model remained the reasonable accuracy of the model with different sample subsets.

Table S1. The results of K-fold validations for the few-shots classification.

Model	Accuracy	AUC	AIC
Final Model	0.95	0.95	8.85
Fold 10	0.91	0.97	34.65
Fold 3	0.87	0.90	111.18

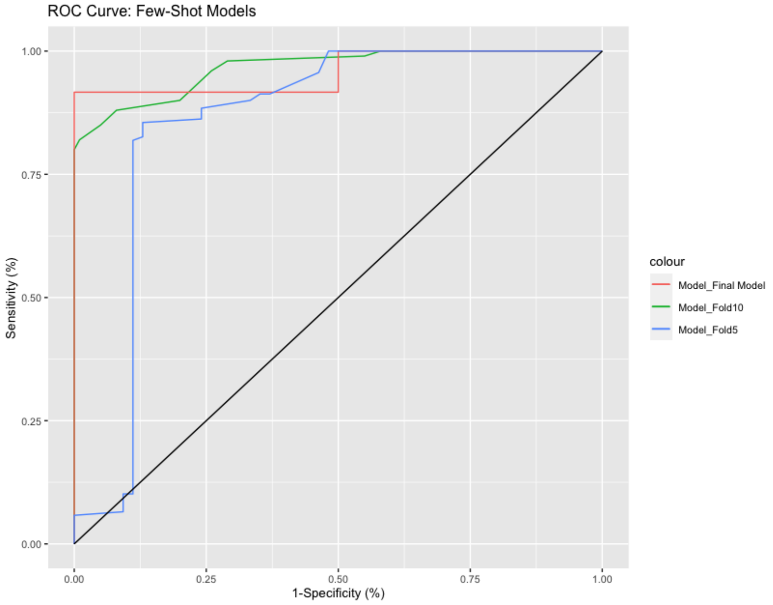

Figure S1. The ROC curve for few-shots classification in K-fold validations.

Radiographic Responses

The radiographic response criteria were modified from MDA criteria which used to distinguish the therapeutic response in bone metastases. The follow-up images, contrast-enhanced CT or MRI before and 3 months after treatment. This imaging-based radiological evaluation standard was believed as the most stringent response criteria of a single bone metastatic lesion. After the MDA evaluation, 16 responders (11 CR cases and 5 PR cases) were observed from selected patients who had received MRgFUS and reported a radiographic response rate of 80% (16 cases /20 patients) with the MDA criteria. The final model with radiographic response showed similar prediction performance with original model with clinical response.

Table S2. Model performances from the radiographic response and clinical response.

Model	Accuracy	AUC	AIC
Radiographic Response	0.90	0.89	108.65
Clinical Response	0.95	0.95	8.85

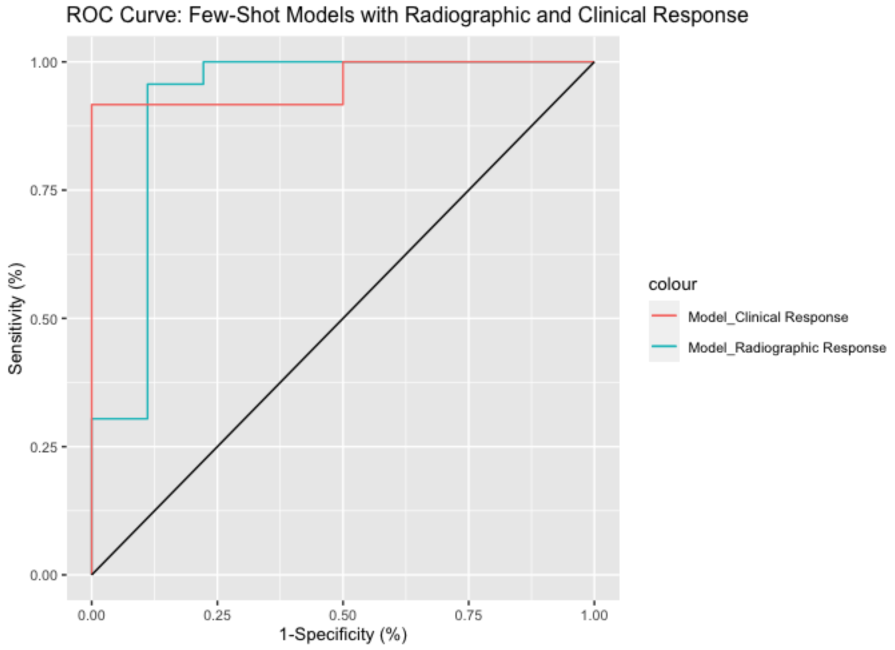

Figure S2. ROC curve of the radiographic response and clinical response.
